# Laparoscopic Management of Spontaneous Gallbladder Perforation in Acalculous Cholecystitis: A Case Series

**DOI:** 10.7759/cureus.73587

**Published:** 2024-11-13

**Authors:** Nikhil M Nagakumar, Ashish Vashistha, Vishal Lakhotia, Aditi Sachdeva, Sourav Panda

**Affiliations:** 1 General Surgery, Max Super Specialty Hospital, New Delhi, IND; 2 General Surgery and Robotics, Max Super specialty Hospital, New Delhi, IND

**Keywords:** acalculous cholecystitis, laparoscopic cholecystectomy, minimal access surgery, spontaneous gallbladder perforation, typhoid-induced acalculous cholecystitis, xanthogranulomatous cholecystitis

## Abstract

Gallbladder perforation in acalculous cholecystitis is rare and has a high mortality rate due to biliary peritonitis and sepsis. Here, we present a case series of successful laparoscopic management of Spontaneous gallbladder perforation in acalculous cholecystitis. In the first case, a 44-year-old male patient presented to the emergency room with a history of three days of fever with chills and acute pain in the abdomen for two days. This was a case of typhoid-induced acalculous cholecystitis with gallbladder perforation, which is a rare clinical entity. The second case was a 62-year-old male, a known diabetic presented with pain abdomen and fever for four days. This was a case of acalculous spontaneous gallbladder perforation. The third case was a 53-year-old female patient with gallbladder perforation in acalculous cholecystitis. Radiological investigations suggested gallbladder perforation which was confirmed on diagnostic laparoscopy in all the cases. Laparoscopic cholecystectomy with peritoneal lavage was performed. All the patients did well postoperatively and were discharged on postoperative day 4 after drain removal. One should be clinically vigilant about gallbladder perforation in a case of acalculous cholecystitis. Minimal access surgery techniques like laparoscopy can be applied to confirm the diagnosis as well as for definitive management.

## Introduction

Gallbladder perforation in association with gallstones, is a well-known entity. However, Spontaneous gallbladder perforation in Acalculous Cholecystitis is uncommon and associated with a high mortality rate of 20%-25% [1]. Acalculous cholecystitis has a higher risk of perforation and necrosis compared to the more typical calculous disease [2]. Pathogenesis involves ischemic necrosis of the gallbladder wall with inflammation. Common predisposing factors for acalculous cholecystitis include infections (typhoid), trauma, immunosuppression, and systemic diseases such as diabetes mellitus and atherosclerosis. Surgical complications of typhoid fever are less common and usually involve the intestine rather than the gallbladder but sometimes we may encounter rarer complications such as acalculous cholecystitis and gallbladder perforation [3,4]. Here, we present three cases of laparoscopic management of spontaneous gallbladder perforation in acalculous cholecystitis.

## Case presentation

Case 1

A 44-year-old male presented to the emergency room (ER) with a three-day history of fever with chills, decreased appetite, and a two-day history of acute pain in the abdomen. He was febrile with 103° F, pulse rate was 106/min, blood pressure was 110/70mmhg and respiratory rate was 20 cycles/min. On physical examination, the abdomen was soft with tenderness and guarding present in the upper abdomen. Blood investigations revealed leukocytosis (15 x 10^9^L) with neutrophilia, increased prothrombin time (16s), and international normalized ratio (INR) (1.4) and the patient tested typhoid IgM positive. Ultrasound (US) of the abdomen showed a distended gallbladder with wall thickening with the normal common bile duct, suggestive of acalculous cholecystitis. Magnetic resonance cholangiopancreatography (MRCP) showed features suggestive of acute cholecystitis and empyema formation with localized perforation in the region of the gallbladder fundus and loculated fluid collection in the gallbladder fossa.

**Figure 1:**
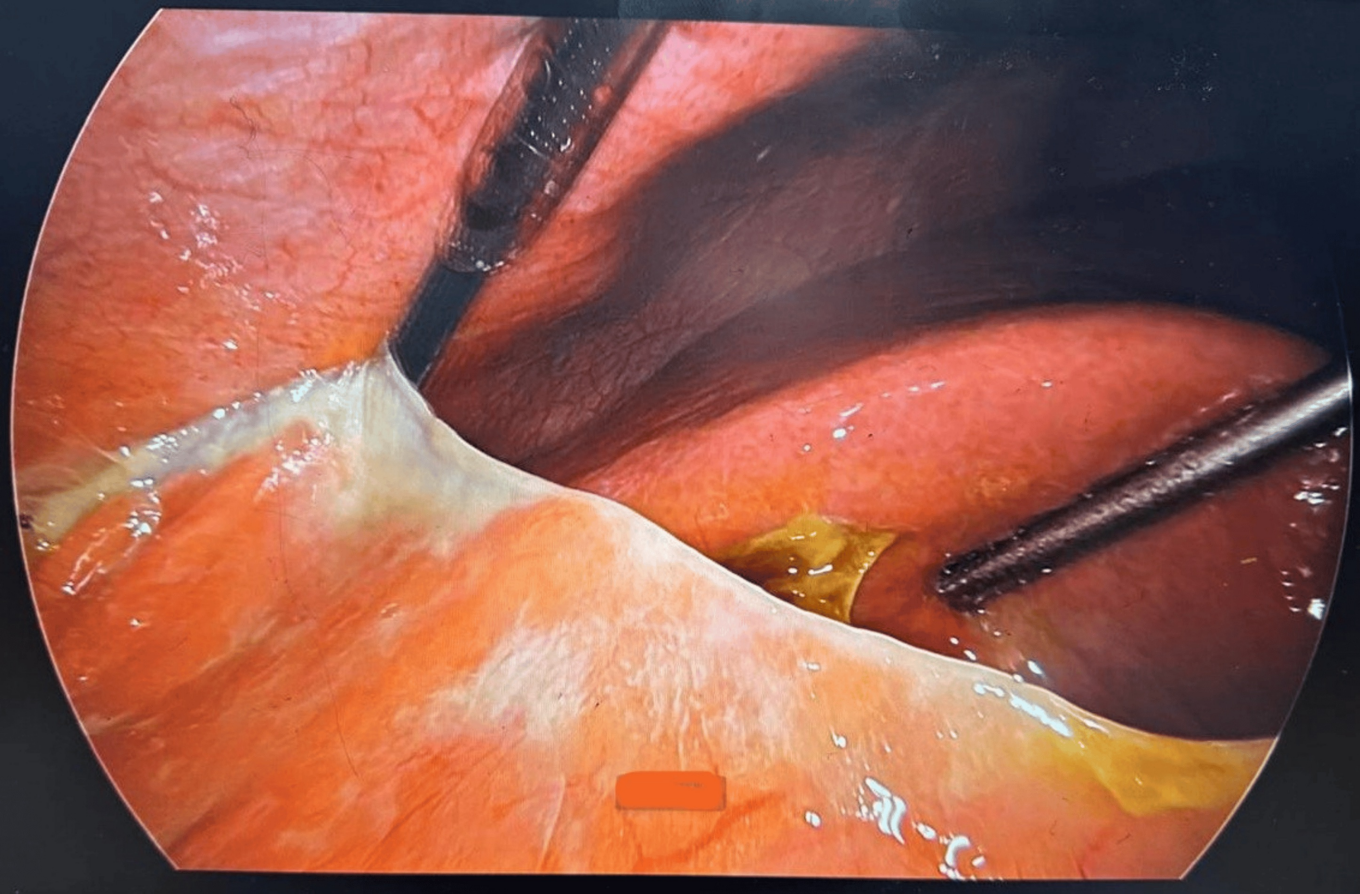
Purulent collection and flakes in Morrison's space and gastrohepatic region with omental and gastric adhesions with anterior abdominal wall

**Figure 2:**
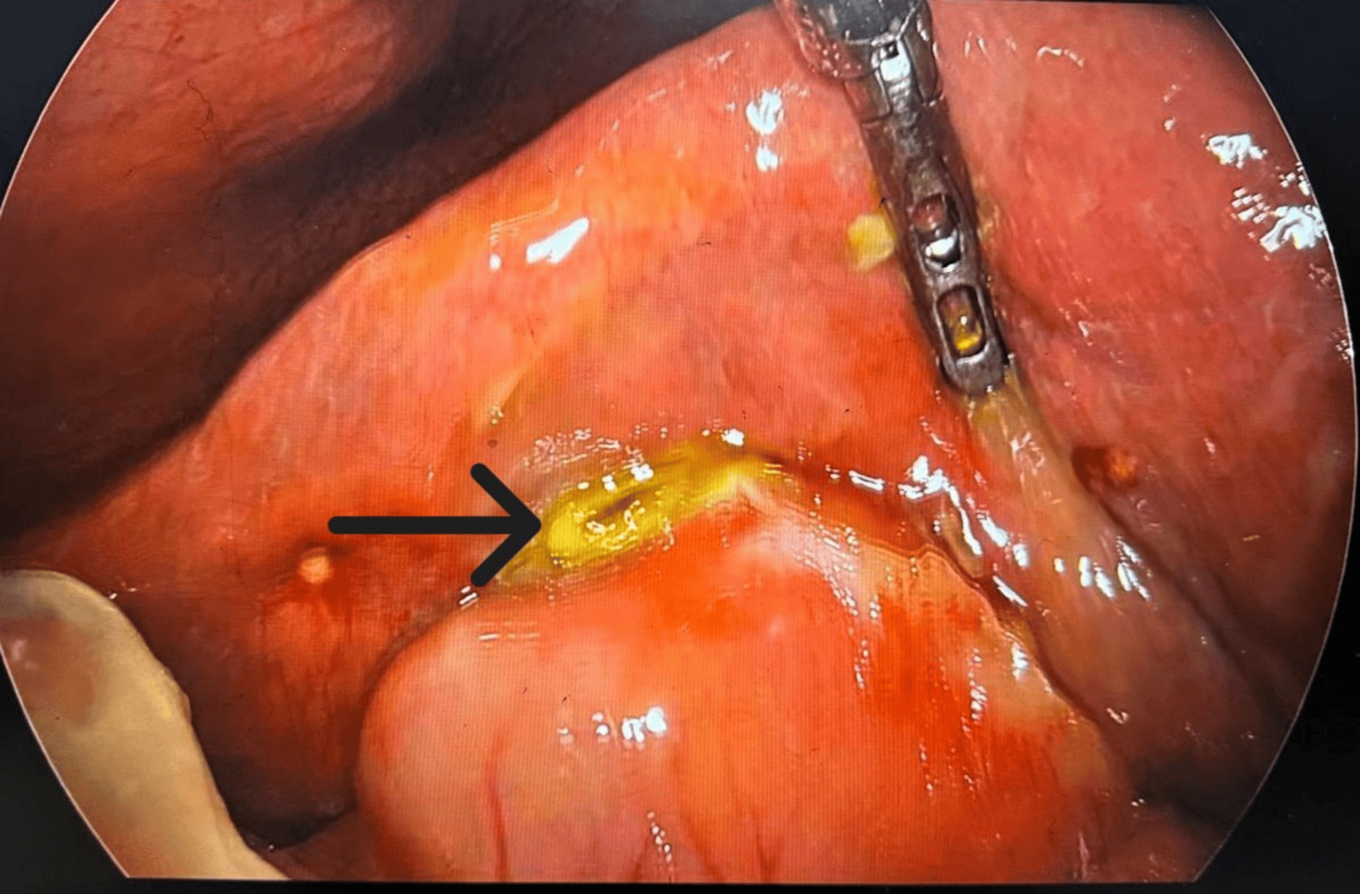
Perforation seen over the fundus of the gallbladder

Based on clinical and radiological findings, a diagnosis of empyema and gallbladder perforation was made, and the patient was taken up for diagnostic laparoscopy. It revealed a purulent collection and flakes in Morrison's space and gastrohepatic region with omental and gastric adhesions with an anterior abdominal wall (Figure 1). On adhesiolysis, perforation was seen over the fundus of the gallbladder (Figure 2). He underwent laparoscopic cholecystectomy with peritoneal lavage. No gallstone was found either in the gallbladder or peritoneal cavity. Postoperatively, the patient was managed with antibiotics, analgesics, and intravenous fluids. An orally soft diet was started on postoperative day 3 (POD 3). The subhepatic drain was removed on POD 4 and he was discharged in stable condition. The patient was stable and comfortable on follow-up (five days after discharge). His gallbladder histopathology report suggested acute or chronic cholecystitis.

Case 2

**Figure 3:**
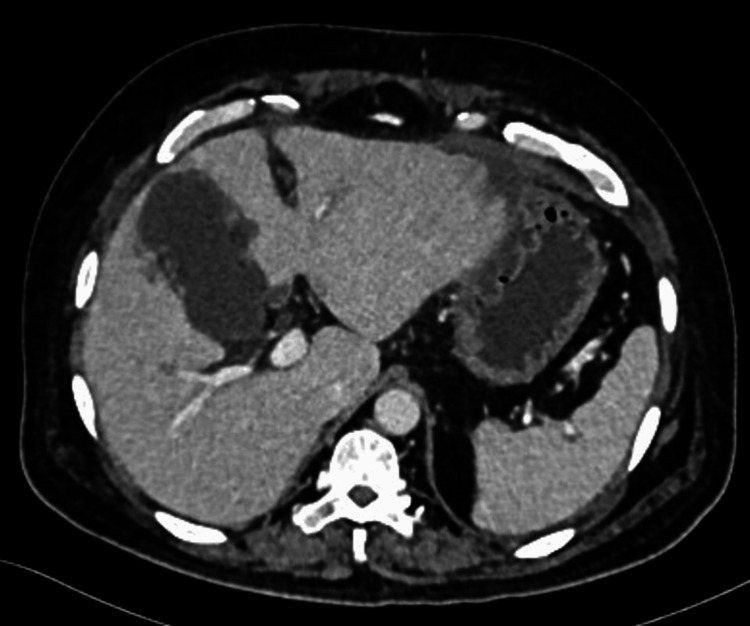
CECT abdomen showing pathological gallbladder having distended lumen with irregular walls which appear dehiscent at places, multiple loculated fluid collections in the pericholecystic and perihepatic subcapsular regions CECT: contrast-enhanced computed tomography

A 62-year-old male presented to the ER with a three-day history of fever, acute pain abdomen, and abdominal distention. On examination, he was febrile with 101.6° F, pulse rate was 103/min, blood pressure was 140/80mmhg, and respiratory rate was 22 cycles/min. On palpation, the abdomen was soft with tenderness present over the right hypochondrium. Blood investigations revealed leukocytosis (27 x 10^9^L) with neutrophilia, increased prothrombin time (17s) and INR (1.6), and increased levels of C-Reactive Protein (240mg/L). US abdomen showed partial distention of the gallbladder with minimal pericholecystic wall edema and, minimal ascites and right pleural effusion. Contrast-enhanced computed tomography (CECT) abdomen showed a pathological gallbladder showing a distended lumen with irregular walls which appear dehiscent at places, and multiple loculated fluid collections in the pericholecystic and perihepatic subcapsular regions (Figure 3). The radiologist suggested the possibility of a gallbladder perforation based on the CECT findings.

**Figure 4:**
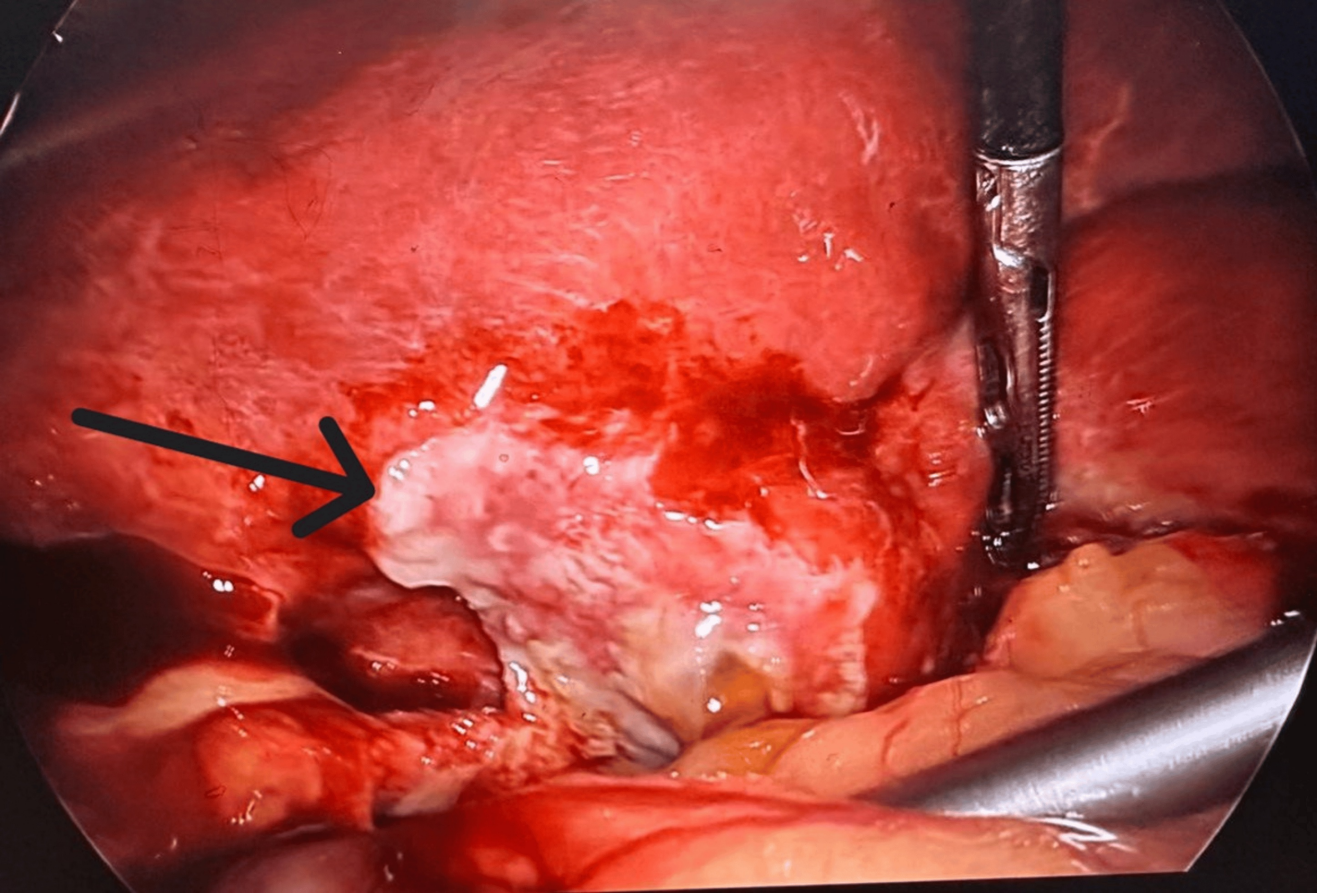
Perforated and sloughed-off gallbladder

The patient was taken up for diagnostic laparoscopy based on clinical and radiological findings. It revealed dense intrabdominal adhesions and purulent collection in the Morrison’s pouch and subdiaphragmatic region. On adhesiolysis and drainage of pus, the gallbladder was seen perforated and sloughed off (Figure 4). He underwent laparoscopic cholecystectomy with peritoneal lavage. No gallstone was found. Postoperatively, the patient was managed with antibiotics, analgesics, and intravenous fluids, followed by oral intake on POD 2. The subhepatic drain was removed on POD 4, and the patient was discharged in satisfactory condition. The patient was stable and comfortable on follow-up (five days after discharge). His gallbladder histopathology report suggested acute necrotizing (gangrenous) cholecystitis.

Case 3

A 53-year-old female presented to us with a five-day history of acute pain abdomen. On examination, she was afebrile at 98° F, pulse rate was 92/min, blood pressure was 130/80mmhg, and respiratory rate was 19 cycles/min. On palpation, the abdomen was soft with tenderness present over the right hypochondrium. Blood investigations revealed leukocytosis (12 x 10^9^L) with neutrophilia and raised total serum bilirubin (2.5 mg/dL). US abdomen showed pathological gallbladder with distended lumen and diffuse wall thickening without obvious calculi. The above features in the US were suggestive of acalculous cholecystitis. MRCP showed focal perforation of the gallbladder in the fundic region and localized perihepatic collection. There was no evidence of cholelithiasis or choledocholithiasis.

**Figure 5:**
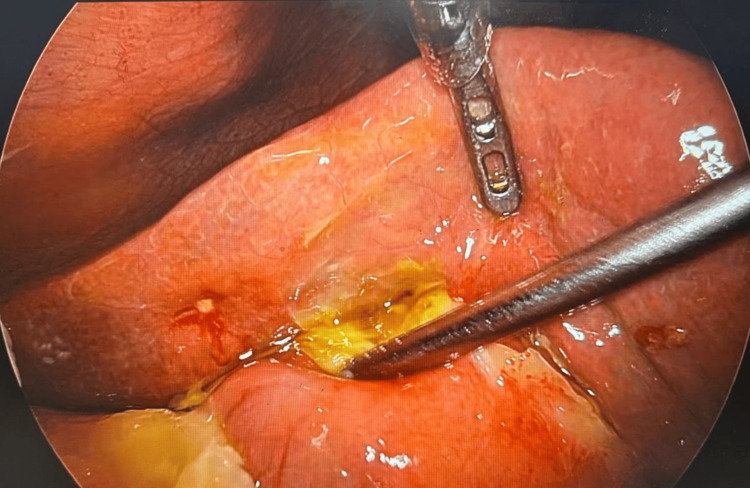
Perforated gallbladder at the fundus

The patient was taken up for diagnostic laparoscopy based on clinical and radiological findings. It revealed dense omental adhesions with gallbladder and purulent collection in the Morrison’s pouch. On adhesiolysis and drainage of pus, the gallbladder was seen perforated on the fundic region (Figure 5). She underwent laparoscopic cholecystectomy with peritoneal lavage. No gallstone was found. The postoperative period was uneventful. The subhepatic drain was removed on POD 4, and the patient was discharged in satisfactory condition. The patient was stable and comfortable on follow-up (five days after discharge). Her gallbladder histopathology report suggested xanthogranulomatous cholecystitis (XGC).

## Discussion

Acute calculous cholecystitis causing gallbladder perforation is a known entity. However, spontaneous gallbladder perforation in acalculous cholecystitis is very rare and associated with a high mortality rate of 20%-25% [1]. Pathogenesis involves ischemic necrosis of the gallbladder wall with inflammation. Predisposing factors for acalculous cholecystitis include infections (typhoid), trauma, immunosuppression, and systemic diseases such as diabetes mellitus and atherosclerosis. Acalculous cholecystitis due to typhoid is very rare. Delay in diagnosing a case of acalculous cholecystitis will usually progress to gangrene or perforation, further increasing the risk of mortality. Surgical complications of typhoid fever are less common and usually involve the intestine than the gallbladder but sometimes we may encounter rarer complications such as acalculous cholecystitis and gallbladder perforation. Most of these cases are found intraoperatively during exploratory laparotomy [5].

XGC is a rare variety of chronic cholecystitis [6]. XGC is a benign but occasionally aggressive disorder resulting from chronic inflammation of the gallbladder wall. There is the presence of xanthogranuloma with predominant lipid-laden macrophages in the gallbladder wall along with fibrosis [7]. Gallbladder perforation due to XGC is a rare clinical entity.

US abdomen is usually the first radiological investigation to assess the gallbladder pathology. In our cases, the US abdomen showed acalculous cholecystitis. However, since patients had peritoneal signs, we got CECT/MRCP of the abdomen, which suggested perforation of the gallbladder.

Since the patients were going into sepsis, we took them for early diagnostic laparoscopy and proceeded with the plan of doing Laparoscopic cholecystectomy. We were able to complete the surgery laparoscopically in all the cases, thus avoiding laparotomy. This minimal-access surgery helped the patients recover faster with no complications. Laparoscopy has the advantage of fast recovery after surgery, less post-operative pain, better cosmesis, and shorter hospital stays [8]. However, good surgical skills are required to manage a perforated gallbladder laparoscopically, as it requires careful adhesiolysis of dense adhesions, dissecting the frozen Callot’s, achieving critical view of safety, adequate peritoneal lavage, and overcoming the poor Endo vision due to acute inflammation. Considering all these surgical hurdles, one should have a low threshold to convert these difficult cases into open surgery [9].

## Conclusions

Minimally invasive techniques, like laparoscopy, can be applied for diagnosing as well as the definitive management of Spontaneous gallbladder perforation in acalculous cholecystitis. This minimal-access surgery helps patients recover faster with minimal or no complications. However, it is necessary to have strong laparoscopic surgical abilities and a low threshold to switch to an open procedure.
